# Hydrazino-methoxy-1,3,5-triazine Derivatives’ Excellent Corrosion Organic Inhibitors of Steel in Acidic Chloride Solution

**DOI:** 10.3390/molecules21060714

**Published:** 2016-06-01

**Authors:** Ayman El-Faham, Sameh M. Osman, Hamad A. Al-Lohedan, Gamal A. El-Mahdy

**Affiliations:** 1 Department of Chemistry, College of Science, King Saud University, P.O. Box 2455, Riyadh 11451, Saudi Arabia; smahmoud@ksu.edu.sa (S.M.O.); hlohedan@ksu.edu.sa (H.A.A.-L.); 2 Chemistry Department, Faculty of Science, Alexandria University, P.O. Box 426, Ibrahimia, Alexandria 12321, Egypt; 3 Advanced Materials Research Chair, Department of Chemistry, College of Science, King Saud University, P.O. Box 2455, Riyadh 11451, Saudi Arabia; 4 Surfactants Research Chair, Department of Chemistry, College of Science, King Saud University, Riyadh 11451, Saudi Arabia; 5 Chemistry Department, Faculty of Science, Helwan University, Helwan 11795, Egypt

**Keywords:** *s*-triazine, hydrazine derivatives, organic corrosion inhibitor, steel, polarization, EIS, adsorption

## Abstract

The corrosion inhibition performance of 2-hydrazino-4,6-dimethoxy-1,3,5-tirazine (DMeHT), 2,4-dihydrazino-6-methoxy-1,3,5-triaizine (DHMeT), and 2,4,6-tridydrazino-1,3,5-triaizne (TH_3_) on steel corrosion in acidic media was examined using electrochemical techniques. The results showed 2,4-Ddihydrazino-6-methoxy-1,3,5-triaizine (DHMeT) gave the best corrosion protection performance among the other hydrazino derivatives even at a low concentration of 25 ppm (95%). The number of hydrazino groups play an important role in the corrosion inhibition, where the two hydrazine groups increased the electrostatic interactions between the protonated tested compounds, the negatively charged steel surface resulted from the adsorption of the chloride anions, and the presence of the methoxy group made the compound more reliable for formation of film protection on the surface of steel through the lone pair of oxygen atoms. Electrochemical Impedance Spectroscopy *(EIS)* measurements suggested that the corrosion process of steel in presence of the hydrazino-*s*-triazine derivatives (TH_3_, DMeHT and DHMeT) were being controlled by the charge transfer reaction. Polarization curves indicated that the examined TH_3_, DMeHT and DHMeT behaved as mixed type inhibitors.

## 1. Introduction

The study of the corrosion phenomena of steel in acidic solution has become predominantly important because of the huge applications in the industry. Organic inhibitors normally inhibit the corrosion of steel by creating a film on the surface of the steel. The efficacy of the inhibitors is dependent on the molecular structure, the chemical composition, and their attractions to the surface of the steel. The efficiency of these compounds are influenced by their electronic structure, aromatic character and the type of functional groups [1,2,3,4].

In recent years, heterocyclic compounds have been extensively studied as organic corrosion inhibitors of steel in acidic solution. Recently, 1,2,4-and 1,2,3-triazole derivatives were reported as a new class of heterocyclic compounds with promising results as organic corrosion inhibitors of steel in 1 M HCl [5,6,7,8,9,10,11,12,13,14,15,16].

1,3,5-Triazine (*s*-triazine) derivatives are another class of heterocyclic compounds and have an excellent potential for the formation of non-covalent bonds, which involve either their nitrogen lone-pairs, their heteroaromatic p-electrons or their σ-backbones [17,18,19,20,21,22]. Recently reported as organic promising corrosion inhibitors of steel in 1 M hydrochloric acid [23], the reported data showed that the corrosion inhibition effect depends on the electronic nature of the groups attached to the triazine moiety [23,24,25,26]. 

**Figure 1:**
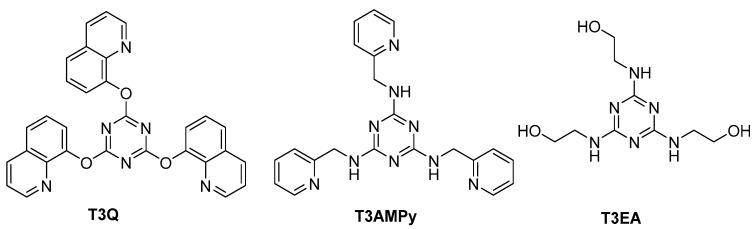
Structure of the new *s*-triazine derivatives as promising organic corrosion inhibitors.

Recently, we reported novel *s*-triazine derivatives as promising organic inhibitors (Figure 1) [27], and the reported results for electrochemical process revealed that, as the nitrogen content increased in the terminal chain, the effeciency for the corrosion protection of steel in acidic solution increased. 

Herein, we report easily prepared compounds with relatively low molecular weight and cheaper materials than the reported ones for triazine deriavtives [23,24,25,26,27] to stress the flexibility and the effect of the number of hydrazino groups along with the methoxy groups that directly attached to the triazine ring for corrosion inhibition of steel in acidic media.

## 2. Results and Discusions 

### 2.1. Synthesis of the Hydrazino-triazine Derivatives

**Figure 2:**
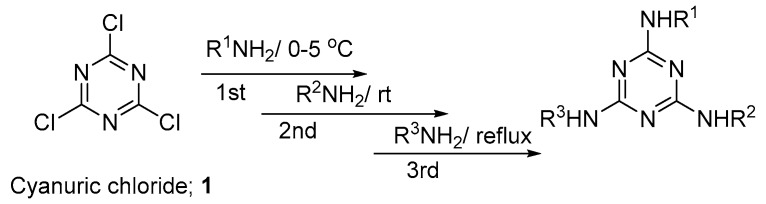
Synthesis of trisubstituted *s*-triazine derivatives from cyanuric chloride.

2,4,6-Trichloro-1,3,5-triazine (cyanuric chloride ) **1** has been known for a long time as an excellent starting material for the synthesis of multitopic molecules [28]. The unique feature of cyanuric chloride is the ability to replace each chlorine atom by any nucleophilic reagent under control of the reaction temperature (Figure 2) [29]. 

**Scheme 1:**
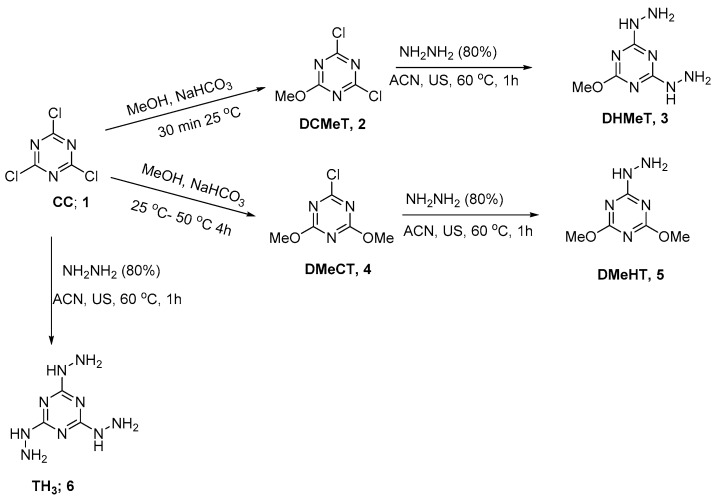
Synthesis of hydrazino-*s*-triazine derivatives.

In this work, cyanuric chloride **1** was first reacted with methanol at 25 °C for 30 min to afford the intermediate 2,4-dichloro-6-methoxy-1,3,5-triazine (DCMeT, **2**) in high yield and purity (Scheme 1). The NMR spectrum (^1^H-NMR and ^13^C-NMR) was in good agreement with the reported data [30]. 

The dichloro derivative (DCMeT) **2** was reacted with hydrazine hydrate using ultrasonic irradiation at 60 °C in acetonitrile as a solvent to afford the product DHMeT **3**; the spectral data was in a good agreement with the reported data (Scheme 1) [31].

The dimethoxy derivatives DMeCT **4** were prepared in the same way for prepation of **2** with a longer reaction time of reaction and heating in methanol for 4 h at 50 °C to afford the chloro-dimethoxy derivatives DMeCT **4** [32]. Reaction of **4** with hydrazine hydrate using ultrasonic irradiation at 60 °C afforded the expected product DMeHT **5** in an excellent yield and purity. 

The trihydrazino TH_3_ **6** was prepared from the reaction of cyanuric chloride **1** with hydrazine hydrate. The reaction was first performed at 0 °C and warmed up to 25 °C and finally sonicated at 60 °C to afford the product in high yield and purity (Scheme 1). The structure of DMeHT **3**, DHMeT **5**, and TH_3_ **6** were confirmed by NMR (^1^H and ^13^C) spectrum and elemental analysis, and were in accordance with the reported data [33,34]. 

### 2.2. Potentiodynamic Polarization Measurements

**Figure 3:**
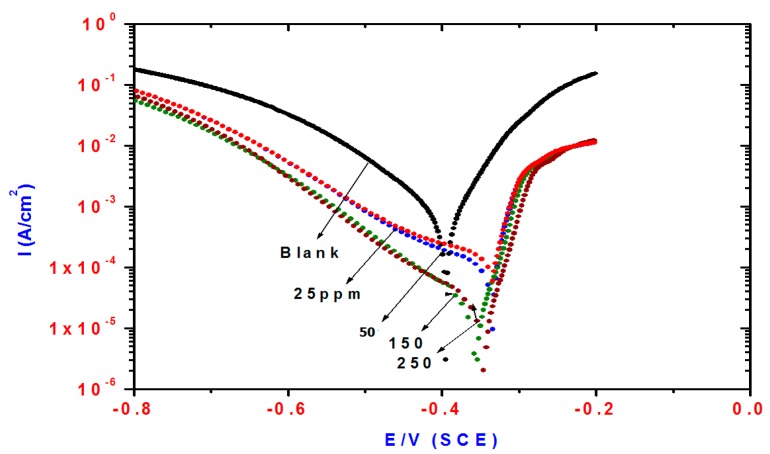
Influence of TH_3_ concentrations on polarization plots of steel electrode in 1 M HCl solution.

**Figure 4:**
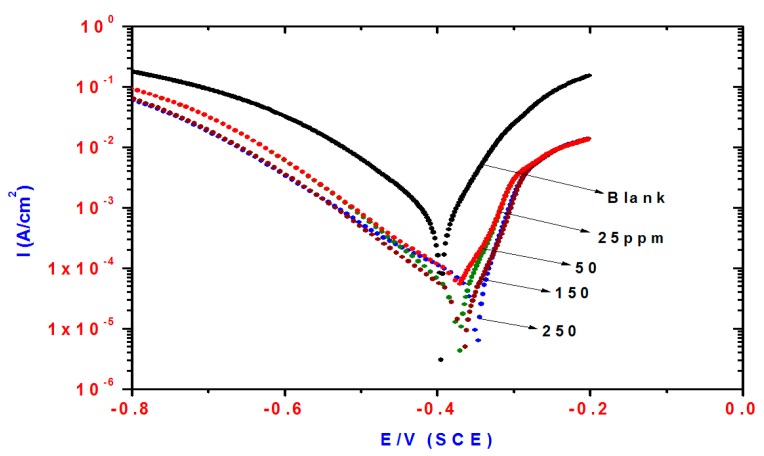
Influence of DMeHT concentrations on polarization plots of steel electrode in 1 M HCl solution.

**Figure 5:**
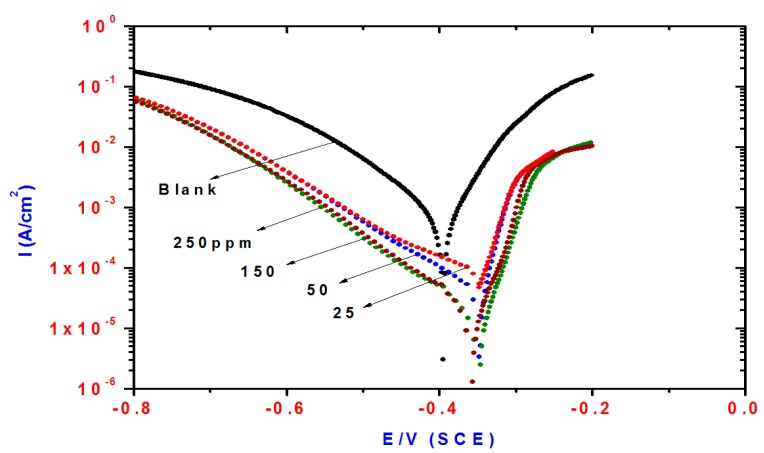
Influence of DHMeT concentrations on polarization plots of steel electrode in 1 M HCl solution.

Cathodic and anodic polarization curves of steel in 1 M HCl solution containing different concentrations of TH_3_ (**6**), DMeHT (**5**) and DHMeT (**3**) are shown in Figure 3, Figure 4 and Figure 5, respectively. The presence of TH_3_, DMeHT and DHMeT lowered the current density of the the anodic and cathodic curves compared with the blank solution. The results may be attributed to adsorption of TH_3_, DMeHT and DHMeT on the steel surface, and hence inhibited the continuation of the corrosion process. This suggests that the TH_3_, DMeHT and DHMeT suppressed the anodic and cathodic reactions by increasing the energy barrier for both processes [35].

As observed from Table 1, the number of hydrazine groups have a great effect on the corrosion inhibition. At high concentrations (250 ppm), the three tested compounds DHMeT, DHMeT, and TH3 have almost the same effect (97.8, 95.2, and 97.8, respectively). While at low concentration (25 ppm and 50 ppm), the dihydrazino DHMeT derivative has the best effect (95.1 and 96.6, respectively). This indicates that the hydrazine groups play an important role in the inhibition efficiency, where the two hydrazine groups increased the electrostatic interactions between the protonated tested compounds and the negatively charged steel surfaces that resulted from the adsorption of the chloride anions, and the presence of the methoxy group makes the compound more reliable for formation of film protection on the surface of steel through the lone pair of oxygen atoms, while increasing the hydrazine group does not improve the efficiency at low concentration as shown in Table 1.

All estimated electrochemical parameters obtained from the extrapolation of the polarization curves are listed in Table 1 for TH_3_, DMeHT and DHMeT. The tested material was labeled as a cathodic or anodic type if the shift in *E_corr_* is >85 mV with respect to *E_corr_* of the blank solution [36]. In addition, the tested material is known as a mixed type inhibitor if the shift in *E_corr_* is <85. It is the clear that the shift in *E_corr_* values is less than 85 mV, suggesting that TH_3_, DMeHT and DHMeT can be classified as a mixed type of inhibitor [37,38]. The inhibition efficiency (*IE*%) that was calculated from polarization can be given as [39,40,41]:

(1) *IE*% = [1 − (*i_corr(inh)_*/*i_corr(uninh)_*)] × 100

where *i_corr(uninh)_* and *i_cor(inh)_* are corrosion current density values in the uninhibited and inhibited solution, respectively. It can be concluded that the higher the TH_3_, DMeHT and DHMeT concentrations, the higher the values of *IE*. The results can be attributed to more adsorption of the inhibitor on steel surface. The diminution of the *I_corr_* values confirms that the TH_3_, DMeHT and DHMeT block the active sites on the steel surface via adsorption of the inhibitor. The predominant corrosion current density value decreased by increasing the inhibitor concentration, showing that TH_3_, DMeHT and DHMeT have corrosion protection performance for the steel corrosion in the acidic chloride-containing environment.

### 2.3. Electrochemical Impedance Spectroscopy (EIS)

**Figure 6:**
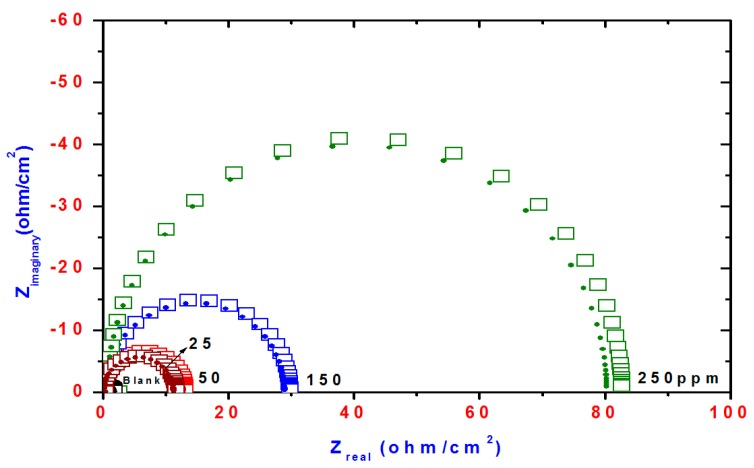
Nyquist plots of steel electrode obtained in 1 M HCl solution and containing various concentrations of TH_3_.

**Figure 7:**
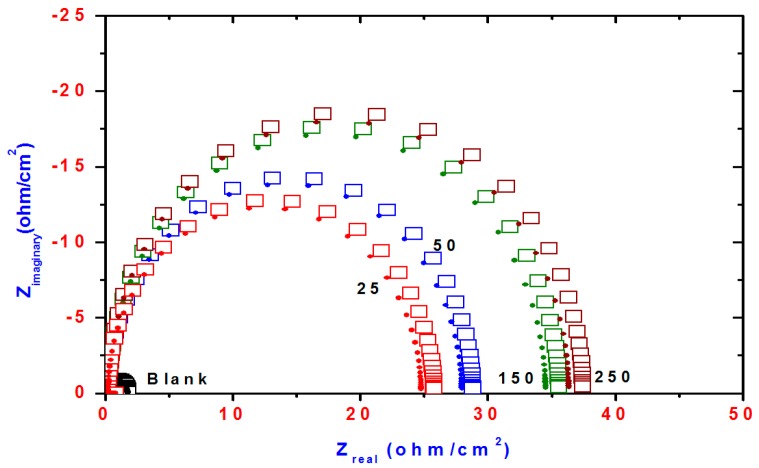
Nyquist plots of steel electrode obtained in 1 M HCl solution and containing various concentrations of DMeHT.

**Figure 8:**
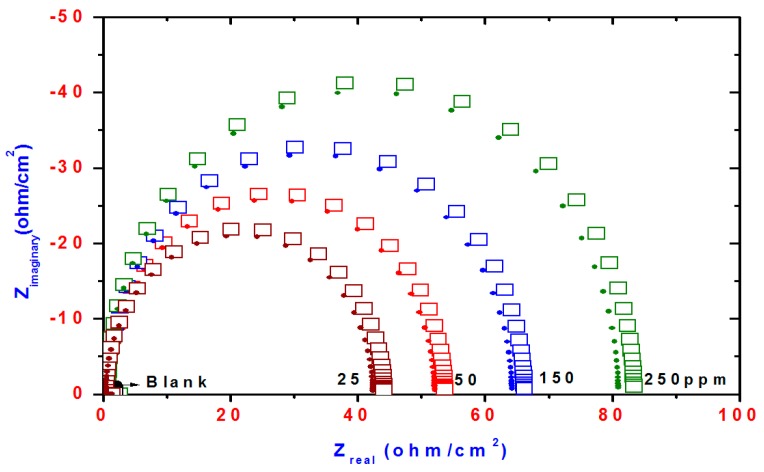
Nyquist plots of steel electrode obtained in 1 M HCl solution and containing various concentrations of DHMeT.

**Figure 9:**
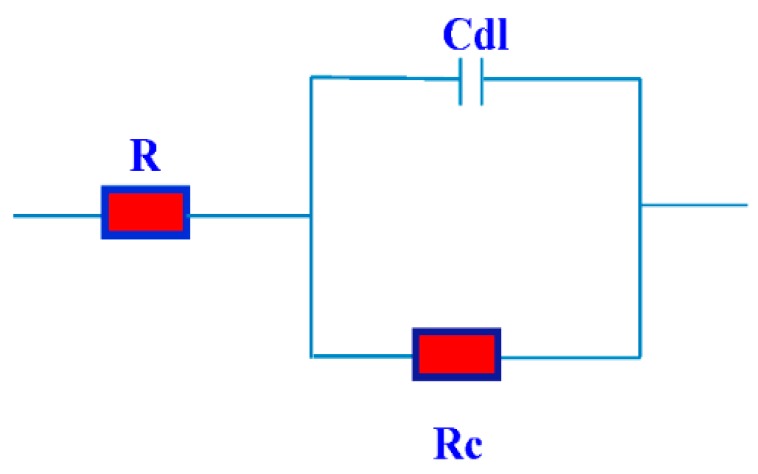
The equivalent used in fitting the electrochemical impedance spectroscopy *(EIS)* data.

Nyquist curves for steel in acidic chloride solution containing different concentrations of TH_3_, DMeHT and DHMeT are shown in Figure 6, Figure 7 and Figure 8, respectively. A single capacitive loop has been observed with an increased diameter with increasing TH_3_, DMeHT and DHMeT concentration. The data shown in Figure 6, Figure 7 and Figure 8 were fitted by an equivalent circuit (*EC*) comprised of solution resistance (*Rs*), charge transfer resistance (*Rct*) in parallel with double layer capacitance (*Cdl*) as shown in Figure 9 [42]. It is composed of (*Rs*), (*Cdl*) and (*Rct*). The values of them are listed in Table 1 for TH_3_, DMeHT and DHMeT. It is clear that the *Rct* values are highly dependent upon the concentration of the tested materials and increase with the increase in TH_3_, DMeHT and DHMeT concentrations. 

The formation of inhibitive films on the steel/solution interface led to an increase in the values of *Rct*. The replacement of pre-adsorbed water molecules (high dielectric constant) on the steel surface by adsorption of TH_3_, DMeHT and DHMeT molecules (with lower dielectric constant) is accompanied by a decrease in the *Cdl* values. *IE*% was estimated from the values of *Rct_(uninh)_* in the uninhibited solution and *Rct_(inh)_* in the inhibited solution as follows [43,44,45]:

(2) *IE*% = [1 − (*Rct_(uninh)_*/*Rct_(inh)_*)] × 100

It is clear that the values of *IE*% increased significantly in the presence of TH_3_, DMeHT and DHMeT, suggesting the protection performance of the tested materials towards the corrosion of steel in the acidic chloride solution. The results can be attributed to an adsorption of TH_3_, DMeHT and DHMeT molecules on the active sites of steel surface, which, in turn, enhanced the high protection performance. It is obvious from the results that the TH_3_, DMeHT and DHMeT inhibited the corrosion ability of steel in the acidic chloride-containing environment even at low concentrations. The calculated values of *IE* presented in Table 1 follow the same trend as those obtained from the polarization results. The results of *IE*% obtained from potentiodynamics and EIS measurements are in good agreement with that reported previously for increasing the *IE*% with an increase in the triazole derivities [15,46].

### 2.4. Adsorption Isotherm

**Figure 10:**
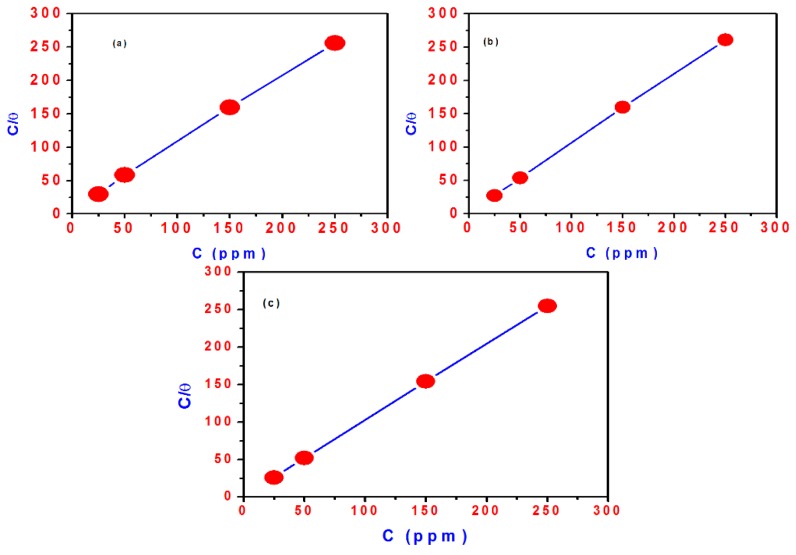
Langmuir adsorption plot of steel in 0.1 M HCl solution containing different concentrations of: (**a**) TH_3_; (**b**) DMeHT; (**c**) DHMeT.

Investigating the adsorption isotherms models is very important for determining the type of interactions of the tested materials with the exposed surface [47]. The experimental data were fitted to the various isotherms [48,49,50,51,52]. The best fit to the collected data of the tested materials is the Langmuir adsorption isotherm (Figure 10), which was described as [53]:

(3) *C_(inh)_*/*θ* = *1/K_ads_* + *C_(inh)_*

where *C_(inh)_* is the inhibitor concentration, Kads is the adsorption equilibrium constant and *θ* is the surface coverage. Plotting of *C_(inh)_/θ* *vs.* *C_(inh)_* exhibited a linear relationship as depicted in Figure 10a–c for TH_3_, DMeHT and DHMeT, respectively. The results indicate that the adsorption of TH_3_, DMeHT and DHMeT on the steel surface follows the Langmuir adsorption isotherm. The constant Kads is related to the standard free energy of adsorption (Δ*G°ads*) by the following equation [54,55]:

(4) Δ*G°ads* = −*RT(ln 55.5Kads)*

where *R* is the gas constant (8.314 J·mol^−1^·K^−1^) and T is the absolute temperature (K). It was established that the existence of electrostatic interaction between charged metal surface and charged organic molecules in the bulk of the solution may be attributed to a small value of ΔG°ads ≤ −20 kJ·mol^−1^ (physical adsorption). The high value of Δ*G°ads* ≥ −40 kJ·mol^−1^ involves charge sharing or charge transfer between the metal surface and organic molecules to form a coordinate type of bond (chemical adsorption) [56,57]. The calculated values of Δ*G°ads* for TH_3_, DMeHT and DHMeT are −34.33, −35.89 and −37.86 kJ·mol^−^^1^, respectively. The estimated values of Δ*G°ads* suggested that the adsorption process of the TH_3_, DMeHT and DHMeT on the steel surface can be labeled as complex interactions, which includes both physical and chemical adsorption [58].

The estimated values of Δ*G°ads* suggested that the adsorption process of the TH_3_, DMeHT and DHMeT on the steel surface can be labeled as complex interactions, which includes both physical and chemical adsorption [59]. 

**Figure 11:**
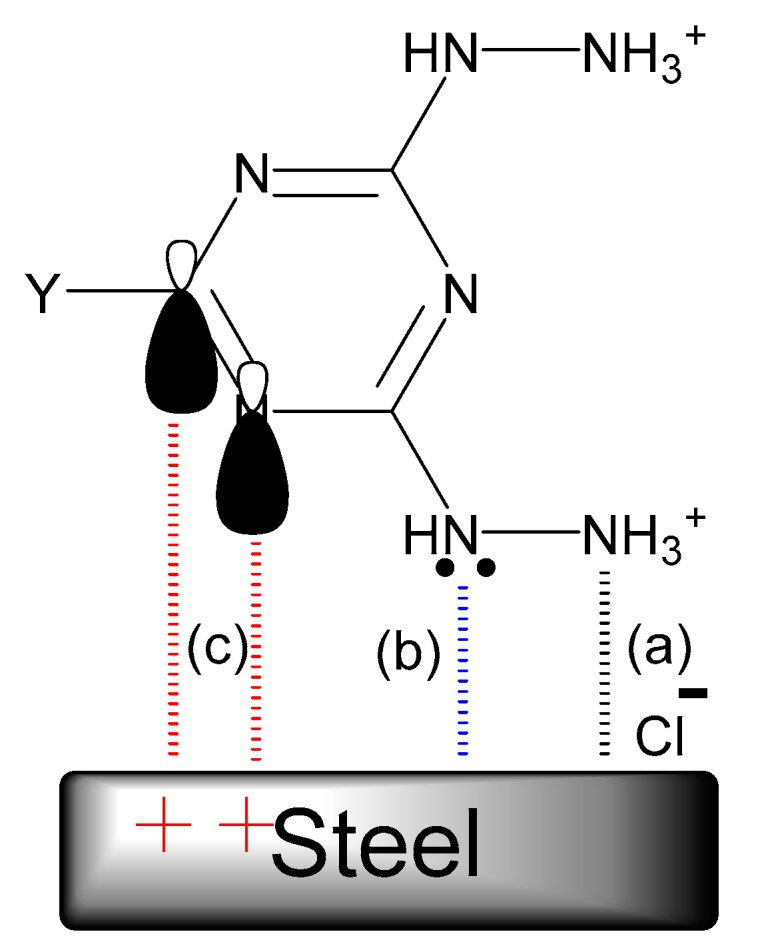
Proposed schematic representation of the adsorption of the hydrazino-1,3,5-triazines on steel in 1.0 M HCl solution: (**a**) physical adsorption; (**b**) chemical adsorption. (**c**) chemical adsorption.

The physical adsorption of the hydrazino-*s*-triazine derivatives occurred between the protonated tested compounds, and the negatively charged steel surface resulted from the adsorption of the chloride anions via electrostatic interactions as shown in Figure 11a, whereas the unshared electron pairs of the nitrogen atoms of the hydrazine group and the triazine ring shared with the empty d-orbital of iron atoms on the steel surface and enhanced the chemical adsorption (Figure 11b). In addition, electron donor-acceptor interactions may also arise between the π-electrons of imine (C=N) groups of 1,3,5 triazine rings and the empty d-orbital of iron atoms (Figure 11c). The adsorption and stability of the adsorbed layer on the steel surface may be attributed to the negative value of Δ*G°ads* [59].

## 3. Experimental Section 

### 3.1. Materials and Methods 

Cyanuric chloride and hydrazine hydrates (80%) were pruchased from Aldrich (Sigma-Aldrich Chemie GmbH, 82024 Taufkirchen, Germany). The solvents used were of HPLC reagent grade. Melting points were determined with a Mel-Temp apparatus and are uncorrected (Sigma-Aldrich Chemie GmbH). The ^1^H-NMR and ^13^C-NMR spectra were recorded on a JEOL 400 MHz spectrometer (JEOL, Ltd., Tokyo, Japan), and the chemical shift values were reported in δ units (ppm). Elemental analyses were performed on a Perkin-Elmer 2400 elemental analyzer (PerkinElmer, Inc., 940 Winter Street, Waltham, MA, USA), and the values found were within ± 0.3% of the theoretical values. The ultrasonic bath was purchased from Selecta (Barcelona, Spain). The purity of the compounds was checked by TLC on silica gel-protected aluminum sheets (Type 60 GF254, Merck, Massachusetts, MA, USA). Tests were performed with steel rods of the following composition (wt %): 0.14% C, 0.57% Mn, 0.21% P, 0.15% S, 0.37% Si, 0.06% V, 0.03% Ni, 0.03% Cr and the remainder Fe. The method of electrode preparation of the working electrode, the reference and the counter electrode are the same as used previously in our studies [27].

The chemical composition, the method of electrode preparation of the working electrode, the reference and the counter electrode are the same as used previously in our studies [27]. 

### 3.2. Electrochemical Measurements

All electrochemical experiments were conducted through the Solartron 1470E system (Potentiostat/Galvanostat) (Indiana, IN, USA) with Solartron 1455A as Frequency Response Analyzer (FRA). Polarization studies were carried out at 1 mV/s scan rate. EIS measurements were executed within the frequency domain 10 kHz to 0.01 Hz using a sine wave of 10 mV amplitude peak to peak. EIS measurements were conducted after 1 h immersion in 1 M HCl solution containing different concentrations of the investigated inhibitors.

### 3.3. Synthesis of 2,4-Dichloro-6-methoxy-1,3,5-triazine (DCMeT, ***2***)

The product was prepared using the reported method [30] and obtained from CH_2_Cl_2_/hexane as a white solid in 98% yield; mp 87–88 °C (Lit. [30]; mp 86–87 °C). ^1^H-NMR (CDCl_3_) δ 3.99 (s, 3H, OCH_3_). ^13^C-NMR (100 MHz, CDCl_3_) δ 54.8 (O*C*H_3_), 168.9, 171.4 (*C*=N, triazine).

### 3.4. Synthesis of 2-Chloro-4,6-dimethoxy-1,3,5-triazine (DMeCT, ***4***)

The product was prepared using the reported method [32] and obtained from CH_2_Cl_2_/hexane as a white solid in 96% yield; mp 73–75 °C [Lit. [32] mp 76–78 °C). ^1^H-NMR (CDCl_3_) δ 3.98 (s, 6H, 2OCH_3_). ^13^C-NMR (100 MHz, CDCl_3_) δ 54.6 (O*C*H_3_), 167.8, 170.2 (triazine moiety). 

### 3.5. General Method for the Synthesis of Hydrazine-1,3,5-triazine Derivatives 

Solution of hydrazine hydrate (20 mL, 80%) in acetonitrile (20 mL) was added to a solution of the chloro derivatives (20 mmol, CC **1**, DCMeT **2**, or DMeCT **4**) in 50 mL acetonitrile at room temperature. The reaction mixture was sonicated at 60 °C for 1 h. The excess solvent and hydrazine hydrate was removed under reduced pressure, and excess of diethyl ether was added to give a slightly pink colored solid which on drying converted to white solid. The solid was collected by filtration, washed with diethyl ether (2 × 50 mL), and finally dried under vacuum to give a pure product in yield 95%–98%. 

*2,4-Dihydrazino-6-methoxy-1,3,5-triazine* (DHMeT, **3**). The product was obtained as a white solid in yield 95%; mp >240 °C. [Lit. [31] 93% yield). IR (KBr, cm^−1^): 3296, 3199, 1584, 1548, 1497. ^1^H-NMR (D_2_O-drop TFA) ppm: δ 3.65 (s, 3H, OCH_3_); ^13^C-NMR (D_2_O-drop TFA) ppm: δ 64.1, 162.3, 162.9. Anal. Calcd for C_4_H_9_N_7_O (171.09): C, 28.07; H, 5.30; N, 57.28; found: C, 28.21; H, 5.41; N, 57.43.

*2-Hydrazino-4,6-dimethoxy-1,3,5-triazine* (DMeHT, **5**). The product obtained as a white solid in yield 96%; m.p 165 °C (dec). IR (KBr, cm^−1^): 3296, 3199, 1584, 1548, 1497. ^1^H-NMR (400 MHz, D_2_O-TFA) δ 3.89 (s, 3H) ppm. ^13^C-NMR (100 MHz, D_2_O-TFA) δ 65.9, 66.1, 162.5, 163.8 ppm. Anal. Calcd for C_5_H_9_N_5_O_2_ (171.16): C, 35.09; H, 5.30; N, 40.92; found: C, 35.22; H, 5.37; N, 41.02.

*2,4,6-Trihydrazino-1,3,5-triazine* (TH_3_, **6**). The product obtained as a white solid in yield 95%; mp >240 °C. [Lit. [33] 93%). IR (KBr, cm^−1^): 3346, 3299, 1580, 1565, 1498. Anal. Calcd for C_3_H_9_N_9_ (171.16): C, 21.05; H, 5.30; N, 73.65; found: C, 21.31; H, 5.42; N, 73.90.

## 4. Conclusions

The three hydrazino-*s*-triazine derivatives TH_3_ **6**, DMeHT **5** and DHMeT **3** are easily prepared from very cheap commercial starting materials and have remarkable protection performance on the corrosion of steel in acidic chloride solution. The number of hydrazine group play an important role in the corrosion inhibition efficiency, where the two hydrazine groups increased the electrostatic interactions between the protonated tested compounds and the negatively charged steel surface that resulted from the adsorption of the chloride anions, and the presence of the methoxy group made the compound more reliable for formation of film protection on the surface of steel through the lone pair of oxygen atoms, while increasing the hydrazine group does not improve the efficiency, especially at low concentration (25 ppm and 50 ppm). Polarization curves indicated that the examined TH_3_, DMeHT and DHMeT were labeled as mixed type corrosion inhibitors. The adsorption of TH_3_, DMeHT and DHMeT onto the steel surface occurred through the nitrogen lone-pairs or its heteroaromatic p-electrons. The protection performance of tested compounds was increased with increasing the number of the hydrazine units in the ring, Dihydrazino derivatives DHMeT showed the best corrosion protection performance among the other hydrazino derivatives even at a low concentration of 25 ppm (95%) The adsorption of TH_3_, DMeHT and DHMeT on the steel surface obeys the Langmuir adsorption isotherm. The calculated values of IE follow the same trend as those obtained from the polarization results.The adsorptions of TH_3_, DMeHT and DHMeT on the steel surface can be explained as complex interactions (both physical and chemical adsorption). 

## Figures and Tables

**Table 1:** Effect of TH_3_, DMeHT and DHMeT concentrations on the inhibition efficiency of steel calculated by electrochemical methods.

Compd.	Polarization Method						EIS Method		
	Conc. (ppm)	*Ba* (mV)	*Bc* (mV)	*E_corr_* (V)	*i_corr_* μA/cm^2^	*IE*%	*Rct* Ohm	*Cdl* (μF/cm^2^)	*IE*%
	Blank	69	120	−0.3955	839	_____	1.80	334	____
**DHMeT; 3**	**25**	**53**	**119**	**−0.3583**	**36**	**95.6**	**43.0**	**110**	**95.1**
	50	54	117	−0.3534	32	96.1	54.0	100	96.6
	150	49	140	−0.3489	22	97.3	66.5	95.0	97.2
	250	50	111	−0.3587	19	98.0	84.0	93.0	97.8
**DMeHT; 5**	**25**	**56**	**164**	**−0.3398**	**59**	**92.9**	**33.0**	**118**	**92.8**
	50	55	160	−0.3498	56	93.3	29.0	108	93.7
	150	58	111	−0.3735	50	94.0	35.8	102	94.0
	250	53	118	−0.3667	34	95.9	37.7	98	95.2
**TH_3_; 6**	**25**	**62**	**230**	**−0.3370**	**124**	**85.0**	**11.6**	**128**	**84.4**
	50	61	227	-0.3380	120	85.6	13.2	122	86.0
	150	43	70	-0.3616	51	93.9	30.0	114	94.0
	250	47	121	-0.3480	19	97.7	83.0	94.0	97.8
